# Predictive value of improvement in the immune tumour microenvironment in patients with breast cancer treated with neoadjuvant chemotherapy

**DOI:** 10.1136/esmoopen-2017-000305

**Published:** 2018-08-30

**Authors:** Wataru Goto, Shinichiro Kashiwagi, Yuka Asano, Koji Takada, Katsuyuki Takahashi, Takaharu Hatano, Tsutomu Takashima, Shuhei Tomita, Hisashi Motomura, Masahiko Ohsawa, Kosei Hirakawa, Masaichi Ohira

**Affiliations:** 1 Department of Surgical Oncology, Osaka City University Graduate School of Medicine, Osaka, Japan; 2 Department of Pharmacology, Osaka City University Graduate School of Medicine, Osaka, Japan; 3 Department of Plastic and Reconstructive Surgery, Osaka City University Graduate School of Medicine, Osaka, Japan; 4 Department of Diagnostic Pathology, Osaka City University Graduate School of Medicine, Osaka, Japan

**Keywords:** tumour microenvironment, breast cancer, immune response, neoadjuvant chemotherapy, tumour-infiltrating lymphocytes

## Abstract

**Background:**

Tumour-infiltrating lymphocytes (TILs) can be used to monitor the immune tumour microenvironment (iTME) and predict treatment response and outcome in breast cancer. We evaluated the prognostic significance of the levels of CD8^+^ TILs and forkhead box protein (FOXP3)-positive TILs before and after neoadjuvant chemotherapy (NAC).

**Patients and methods:**

We examined 136 patients with breast cancer treated with NAC. The number of CD8^+^ TILs and FOXP3^+^ TILs in biopsy specimens and residual tumours was evaluated by immunohistochemistry.

**Results:**

Patients with a high rate of change in the CD8/FOXP3 ratio (CFR) had significantly better recurrence-free survival (RFS) (p<0.001, log-rank). In multivariate analysis, the rates of change in the CD8^+^ TIL levels and the CFR were independent predictors for RFS (HR=2.304, p=0.036 and HR=4.663, p<0.001). In patients with triple-negative and hormone receptor-positive breast cancer, the rate of change in the CFR was an independent predictor for RFS (HR=13.021, p=0.002 and HR=4.377, p=0.003).

**Conclusion:**

Improvement in the iTME following NAC is correlated with good outcome. The rate of change in the CFR may be a useful biomarker to predict prognosis of patients treated with NAC.

**Key questions**

**What is already known about this subject?**

- Recently, the importance of regulating and improving the immune tumour microenvironment (iTME) has been reported to play an important role in predicting outcomes.
- Tumour-infiltrating lymphocytes (TILs) can be used to monitor the iTME and predict treatment response and outcome in breast cancer.
- CD8/FOXP3 ratio (CFR) in biopsy specimens before neoadjuvant chemotherapy (NAC) is a useful biomarker to predict treatment response to chemotherapy.

**What does this study add?**

- The predictive value of changes in lymphocytic subpopulations after NAC in all breast cancer subtypes has not been discussed sufficiently.
- The present study investigated the clinical significance and value of changes in the levels of CD8^+^ TILs and FOXP3^+^ TILs and the CFR before and after NAC in all breast cancer subtypes.
- To our knowledge, this is the first study to demonstrate the prognostic role of changes in CD8^+^ TIL levels, FOXP3^+^ TIL levels and the CFR in patients failing to achieve pathological complete response following NAC in all breast cancer subtypes.

**How might this impact on clinical practice?**

- These results suggest that by further evaluating the changes in other TILs, such as programmed death-1-positive TILs, along with those in CD8^+^ TILs and FOXP3^+^ TILs in patients treated with NAC, more accurate identification of patient-specific immune mechanisms and prediction of prognosis may be possible.
- Improvement in the iTME following NAC is correlated with good outcome.
- The rate of change in the CFR may be a useful biomarker to predict prognosis of patients treated with NAC.

## Introduction

Neoadjuvant chemotherapy (NAC) is the gold standard of care for breast cancer and increases the options for breast-conserving surgery.^1–3^ Pathological complete response (pCR) after NAC is currently acknowledged as an indicator of good outcome, especially in triple-negative breast cancer (TNBC) and human epidermal growth factor receptor 2 (HER2)-enriched breast cancer (HER2BC).^4^ One previous study indicated that residual cancer cells after NAC may be more aggressive or have enhanced metastatic potential.^5^ However, some patients who fail to achieve pCR after NAC have a relatively good outcome. Therefore, novel prognostic markers in residual tumours are needed to identify high-risk patients.

Recently, the importance of regulating and improving the immune tumour microenvironment has been reported to play an important role in predicting outcomes.^6 7^ Tumour-infiltrating lymphocytes (TILs) can be used to monitor the tumour microenvironment and are important in predicting treatment efficacy and clinical outcomes in many types of cancer, including breast cancer.^8–10^ Various cells of the immune system can play varying roles in tumour progression; for instance, cytotoxic T cells (CD8^+^ T cells), natural killer cells, dendritic cells and macrophages are associated with improved clinical outcomes, whereas regulatory T (Treg) cells and myeloid-derived suppressor cells suppress antitumour immunity.^11^ Specific TIL subsets, such as CD3^+^, CD8^+^ and forkhead box protein 3 (FOXP3)-positive TILs, have been reported to be clinically significant and reliable in predicting treatment response.^12–14^ In addition, since Treg cells suppress the induction of cytotoxic T cells in response to cancer cells, the CD8/FOXP3 ratio (CFR) has been reported to be associated with high pCR rates.^15 16^ We have also suggested that the CFR in biopsy specimens before NAC is a useful biomarker to predict treatment response to chemotherapy in TNBC and HER2BC.^17^

Chemotherapy enhances the immune activity or the reversal of immunosuppression.^7 18^ Some studies revealed that changes in the levels of CD8^+^ or FOXP3^+^ TILs induced by chemotherapy can be used as a prognostic marker in aggressive breast cancer subtypes, such as TNBC.^15 19 20^ However, the predictive value of changes in lymphocytic subpopulations after NAC in all breast cancer subtypes has not been discussed sufficiently.

The present study investigated the clinical significance and value of changes in the levels of CD8^+^ TILs and FOXP3^+^ TILs and the CFR before and after NAC in all breast cancer subtypes. To our knowledge, this is the first study to demonstrate the prognostic role of changes in CD8^+^ TIL levels, FOXP3^+^ TIL levels and the CFR in patients failing to achieve pCR following NAC in all breast cancer subtypes.

## Patients and methods

### Ethics

This study was conducted at the Osaka City University Graduate School of Medicine, Osaka, Japan according to the Reporting Recommendations for Tumour Marker Prognostic Studies guidelines and following a retrospectively written research proposal, pathological evaluation and statistical plan.^21^ This study conformed to the provisions of the Declaration of Helsinki. All patients were informed of the investigational nature of this study and provided their written informed consent.

### Patient background

A total of 214 patients with resectable, early-stage, primary infiltrating ductal breast cancer who were treated with NAC between 2007 and 2015 were included. Tumour stage and T and N factors were stratified based on the TNM Classification of Malignant Tumours, The Union for International Cancer Control Seventh Edition.^22^ Tumours were classified into subtypes according to the immunohistochemical expression of oestrogen receptor (ER), progesterone receptor (PgR), HER2 and Ki-67.

### Neoadjuvant therapy regimen and surgery

All patients received a standardised NAC protocol consisting of four courses of FEC100 (500 mg/m^2^ fluorouracil, 100 mg/m^2^ epirubicin and 500 mg/m^2^ cyclophosphamide) every 3 weeks, followed by 12 courses of 80 mg/m^2^ paclitaxel administered weekly.^23 24^ Patients with HER2BC were additionally administered weekly (2 mg/kg) or tri-weekly (6 mg/kg) with trastuzumab during paclitaxel treatment.^25^

### Clinical end points

Therapeutic antitumour effects were assessed according to the Response Evaluation Criteria in Solid Tumors criteria.^26^ The pCR was defined as the complete disappearance of the invasive compartment of the lesion with or without intraductal components, including the lymph nodes.^27^ Overall survival (OS) was the period from the date of primary surgery to the date of death from any cause. Recurrence-free survival (RFS) was defined as the period from the date of primary surgery until the date of disease recurrence.

### Immunohistochemistry

All patients underwent a core needle biopsy prior to NAC and curative surgery involving mastectomy or conservative surgery with axillary lymph node dissection after NAC. Immunohistochemical studies were performed as described previously.^17^ Primary monoclonal antibodies directed against ER (clone 1D5, dilution 1:80; Dako), PgR (clone PgR636, dilution 1:100; Dako), HER2 (HercepTest; Dako), Ki-67 (clone MIB-1, dilution 1:00; Dako), CD8 (clone C8/144B, dilution 1:100; Dako) and FOXP3 (clone 236A/E7, dilution 1:100; Abcam, Cambridge, UK) were used.

### Immunohistochemical scoring

Immunohistochemical scoring was performed by two breast pathologists (MOhs and YK). The cut-offs for ER and PgR positivity were both >0% positive tumour cells with nuclear staining. Tumours with 3+HER2 on immunohistochemical staining were considered to have HER2 overexpression, tumours with 2+HER2 were analysed further by fluorescence in situ hybridisation and tumours with *HER2/Centromere 17* ≥2.0 were also considered to exhibit HER2 overexpression.^28^ Tumours with ≥14% Ki-67 nuclear staining were determined to be positive.^29^

**Figure 1:**
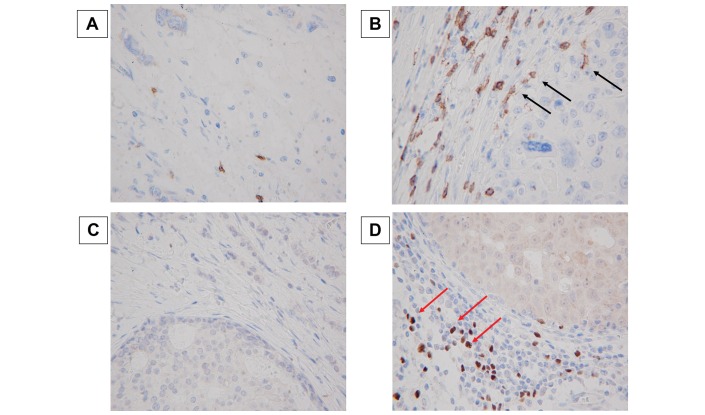
Immunohistochemical determination of tumour-infiltrating CD8^+^ and FOXP3^+^ lymphocytes. Representative immunohistochemical pictures of low (A) and high (B) CD8^+^ staining (400×). Representative immunohistochemical pictures of low (C) and high (D) FOXP3^+^ staining (400×). Black arrow points to CD8^+^ lymphocytes, and red arrow points to FOXP3^+^ lymphocytes). Goto *et al*.^17^ FOXP3, forkhead box protein 3.

The assessment of unstained TILs was based on the criteria described by Salgado and colleagues.^11^ TILs were evaluated within the stromal compartment close to the invasive tumour, and the proportion of stromal TILs was reported. In this study, unstained TILs were considered high if TILs occupied >10% of the stroma area and low when TILs occupied ≤10%. To evaluate CD8 and FOXP3 expression, four fields of view (FOV) in darkly stained areas were selected, and the number of TILs in the stroma surrounding the stained cancer cells in each FOV was measured microscopically at 400× magnification (figure 1). The mean number of CD8^+^ or FOXP3^+^ lymphocytes in each FOV was counted. The CFR was defined as the number of CD8^+^ TILs divided by the number of FOXP3^+^ TILs.

### Statistical analyses

Statistical analysis was performed using the JMP13 software programme (SAS Institute). The associations between levels of CD8^+^ TILs and FOXP3^+^ TILs and clinicopathological variables were analysed using Χ^2^ tests or Fisher’s exact tests, as appropriate. OS and RFS were estimated using the Kaplan-Meier method and compared using the log-rank test. Univariate and multivariate HRs were computed for the study parameters with 95% CIs using a Cox proportional hazards model and used in a backward stepwise method for variable selection in multivariate analyses. A p value <0.05 was considered significant.

## Results

### CD8^+^ TILs and FOXP3^+^ TILs before NAC and outcome

Among 214 patients, 78 (36.4%) patients achieved pCR. Therefore, 136 (63.6%) patients with residual tumour after NAC were included in the study. Except for one patient who had insufficient tissue for immunohistochemical staining, the baseline levels of CD8^+^ TILs and FOXP3^+^ TILs and the CFR before NAC are presented in online supplementary table 1. Unstained TILs (%) ranged from 0 to 90 (mean, 16; median, 18; SD, 5). CD8^+^ TILs ranged from 0 to 138 (mean, 38; median, 36; SD, 22). FOXP3^+^ TILs ranged from 0 to 55 (mean, 14; median, 17; SD, 11). In each breast cancer subtype, the proportion of unstained TILs tended to be higher in patients with TNBC than in other breast cancer subtypes (p=0.055), but there was no relationship between CD8^+^ TILs and FOXP3^+^ TILs and breast cancer subtypes (p=0.838 and p=0.570, respectively). The cut-off levels for high or low infiltration were based on the mean number of infiltrating cells per field as follows: CD8^+^ TILs, 38; FOXP3^+^ TILs, 14 and CFR, 3.1. Ki-67 was significantly higher in patients with low levels of FOXP3^+^ TILs (p=0.016). The proportion of unstained TILs was significantly higher in patients with high levels of CD8^+^ TILs (p=0.015), low levels of FOXP3^+^ TILs (p=0.003) and high levels of CFR (p<0.001). There were significant positive correlations among the levels of CD8^+^ TILs and FOXP3^+^ TILs and the CFR (CD8^+^ vs FOXP3^+^: p<0.001, CD8^+^ vs CFR: p<0.001, FOXP3^+^ vs CFR: p<0.001). No correlations between any other tested clinicopathological parameter and the levels of CD8^+^ TILs and FOXP3^+^ TILs and the CFR were found. RFS was significantly longer in the high CFR group than in the low CFR group (p=0.013), but OS was not significantly different (p=0.054, log-rank) (online supplementary figure 1).

10.1136/esmoopen-2017-000305.supp1 Supplementary file 1

10.1136/esmoopen-2017-000305.supp2 Supplementary file 2

### CD8^+^ TILs and FOXP3^+^ TILs after NAC and outcome

Except for six patients who had either insufficient tissue or no available tissue for immunohistochemical staining, the levels of CD8^+^ TILs and FOXP3^+^ TILs and the CFR after NAC are presented in online supplementary table 2. Unstained TILs (%) ranged from 0 to 94 (mean, 23; median, 29; SD, 7). CD8^+^ TILs ranged from 0 to 141 (mean, 42; median, 53; SD, 23). FOXP3^+^ TILs ranged from 0 to 67 (mean, 7; median, 6; SD, 6). In each breast cancer subtype, there was no relationship between unstained TILs, CD8^+^ TILs, FOXP3^+^ TILs and breast cancer subtypes (p=0.168, p=0.772 and p=0.579, respectively). The cut-off levels for high or low infiltration of the residual tumour after NAC were as follows: CD8^+^ TILs, 42; FOXP3^+^ TILs, 7 and CFR, 7.0. Younger patients (≤56 years) had significantly higher levels of CD8^+^ (p=0.035) and FOXP3^+^ (p=0.024) TILs than older patients (>56 years). The partial response (PR) rate was significantly higher in the high CFR group than in the low CFR group (p=0.012). The proportion of unstained TILs was significantly higher in patients with high levels of CD8^+^ TILs (p<0.001) and high levels of FOXP3^+^ TILs (p<0.001), but there was no significant correlation between unstained TILs and the CFR (p=0.364). The high CD8^+^ TILs group had significantly better RFS and OS than the low CD8^+^ TILs group (p=0.001, p=0.017, log-rank, respectively). The low FOXP3^+^ TILs group had significantly better RFS and OS than the high FOXP3^+^ TILs group (p=0.006, p=0.005, log-rank, respectively). A high CFR was also significantly correlated with better RFS and OS (p<0.001, both end points) (online supplementary figure 2).

10.1136/esmoopen-2017-000305.supp3 Supplementary file 3

10.1136/esmoopen-2017-000305.supp4 Supplementary file 4

### Changes in CD8^+^ TILs and FOXP3^+^ TILs before and after NAC and their association with prognosis

**Figure 2:**
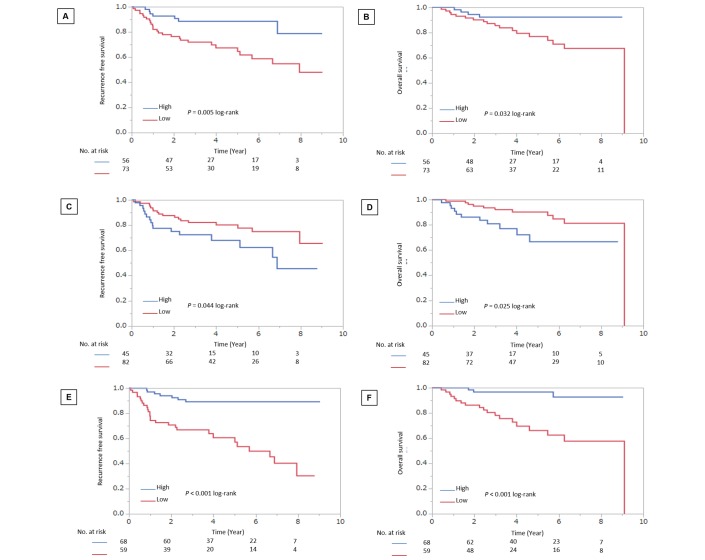
Analysis of the rate of changes in CD8^+^ TILs, FOXP3^+^ TILs and the CFR and RFS and OS in patients with all breast cancer subtypes. Patients with a high rate of change in CD8^+^ TIL levels had significantly better RFS (A) and OS (B) than those with a low rate of change (p=0.005, p=0.032, log-rank, respectively). Patients with a low rate of change in FOXP3^+^ TIL levels had significantly better RFS (C) and OS (D) than those with a high rate of change (p=0.044, p=0.025, log-rank, respectively). Patients with a high rate of change in the CFR also had significantly better RFS (E) and OS (F) than those with a low rate of change (p<0.001, log-rank, both end points). Goto *et al*.^17^ CFR, CD8/FOXP3 ratio; FOXP3, forkhead box protein 3; OS, overall survival; RFS, recurrence-free survival; TIL, tumour-infiltrating lymphocyte.

The mean rates of change in CD8^+^ TIL levels, FOXP3^+^ TIL levels and the CFR before and after NAC were as follows: CD8^+^ TILs, 1.2; FOXP3^+^ TILs, 0.5 and CFR, 2.3. Of 129 patients, 56 (43.4%) had a high rate of change in CD8^+^ TILs, 82 (63.6%) had a low rate of change in FOXP3^+^ TILs and 68 (52.7%) had a high rate of change in the CFR. In addition, 62 (48.1%) patients had a high rate of change in unstained TILs. Younger patients (≤56 years) were significantly more likely to have a high rate of change in CD8^+^ TIL levels than older patients (>56 years) (p=0.013). Patients with TNBC had a significantly higher rate of change in FOXP3^+^ TIL levels than patients with other subtypes (p=0.014) (table 1). Patients with a high rate of change in unstained TILs were significantly higher in patients with a high rate of change in CD8^+^ TIL levels (p<0.001) and a high rate of change in FOXP3^+^ TILs (p=0.003), but there was no significant correlation between the rate of change in unstained TILs and the CFR (p=0.479). Patients with a high rate of change in CD8^+^ TIL levels had significantly better RFS and OS than those with a low rate of change (p=0.005, p=0.032, log-rank, respectively). Patients with a low rate of change in FOXP3^+^ TIL levels had significantly better RFS and OS than those with a high rate of change (p=0.044, p=0.025, log-rank, respectively). Patients with a high rate of change in the CFR also had significantly better RFS and OS than those with a low rate of change (p<0.001, log-rank, both end points) (figure 2).

**Table 1:** Correlation between clinicopathological features and CD8^+^ TILs change, FOXP3^+^ TILs change and the CD8/FOXP3 ratio

Parameters	CD8^+^ TILs change		P values	FOXP3^+^ TILs change		P values	CD8/FOXP3 change		P values
	Low (n=73)	High (n=56)		Low (n=82)	High (n=45)		Low (n=59)	High (n = 68)	
Age at operation									
≤56 years	29 (39.7%)	35 (62.5%)	0.013	37 (45.1%)	26 (57.8%)	0.197	32 (54.2%)	31 (45.6%)	0.376
>56 years	44 (60.3%)	21 (37.4%)		45 (54.9%)	19 (42.2%)		27 (45.8%)	37 (54.4%)	
Tumour size									
≤2 cm	12 (16.4%)	4 (7.1%)	0.177	11 (13.4%)	5 (11.1%)	0.787	8 (13.6%)	8 (11.8%)	0.794
>2 cm	61 (83.6%)	52 (92.9%)		71 (86.6%)	40 (88.9%)		51 (86.4%)	60 (88.2%)	
Lymph node status									
Negative	18 (24.7%)	20 (35.7%)	0.18	24 (29.3%)	13 (28.9%)	0.964	13 (22.0%)	24 (35.3%)	0.119
Positive	55 (75.3%)	36 (64.3%)		58 (70.7%)	32 (71.1%)		46 (78.0%)	44 (64.7%)	
Ki-67									
≤14%	36 (49.3%)	26 (46.4%)	0.859	43 (52.4%)	17 (37.8%)	0.138	28 (47.5%)	32 (47.1%)	0.964
>14%	37 (50.7%)	30 (53.6%)		39 (47.6%)	28 (62.2%)		31 (52.5%)	36 (52.9%)	
Intrinsic subtype									
TNBC	22 (30.2%)	16 (28.6%)	0.942	18 (21.9%)	20 (44.4%)	0.014	21 (35.6%)	17 (25.0%)	0.426
HER2BC	9 (12.3%)	8 (14.3%)		10 (12.2%)	7 (15.6%)		7 (11.9%)	10 (14.7%)	
HRBC	42 (57.5%)	32 (57.1%)		54 (65.9%)	18 (40.0%)		31 (52.5%)	41 (60.3%)	
Pathological response									
Non-PR	13 (17.8%)	5 (8.9%)	0.202	10 (12.2%)	8 (17.8%)	0.431	12 (20.3%)	6 (8.8%)	0.071
PR	60 (82.2%)	51 (91.1%)		72 (87.8%)	37 (82.2%)		47 (79.7%)	62 (91.2%)	
TILs (%) change									
Low	53 (72.6%)	14 (25.0%)	<0.001	50 (61.0%)	15 (33.3%)	0.003	28 (47.5%)	37 (54.4%)	0.479
High	20 (27.4%)	42 (75.0%)		32 (39.0%)	30 (66.7%)		31 (52.5%)	31 (45.6%)	
CD8^+^TILs									
Low	Not determined	Not determined		51 (62.2%)	22 (48.9%)	0.189	39 (66.1%)	34 (50.0%)	0.075
High				31 (37.8%)	23 (51.1%)		20 (33.9%)	34 (50.0%)	
FOXP3^+^ TILs									
Low	51 (69.9%)	31 (57.4%)	0.189	Not determined	Not determined		21 (35.6%)	61 (89.7%)	
High	22 (30.1%)	23 (42.6%)					38 (64.4%)	7 (10.3%)	<0.001
CD8^+^/FOXP3^+^									
Low	39 (53.4%)	21 (37.5%)	0.073	21 (25.6%)	38 (84.4%)	<0.001	Not determined	Not determined	
High	34 (46.6%)	35 (62.5%)		61 (74.4%)	7 (15.6%)				

FOXP3, forkhead box protein; HER2BC, human epidermal growth factor receptor 2-enriched breast cancer; HRBC, hormone receptor-positive breast cancer; PR, partial response; TIL, tumour-infiltrating lymphocyte; TNBC, triple-negative breast cancer.

In univariate analysis, pathological response (HR=6.33, 95% CI 2.893 to 13.13, p<0.001), the rate of change in CD8^+^ TIL levels (HR=3.114, 95% CI 1.430 to 7.773, p=0.003) and the CFR (HR=5.612, 95% CI 2.581 to 14.001, p<0.001) were found to be favourable prognostic factors. The rate of change in unstained TILs was not a significant prognostic factor (HR=1.276, 95% CI 0.656 to 2.536, p=0.473). Multivariate analysis showed that PR (HR=5.260, 95% CI 2.373 to 11.145, p<0.001), a high rate of change in CD8^+^ TIL levels (HR=2.304, 95% CI 1.052 to 5.776, p=0.036) and the CFR (HR=4.663, 95% CI 2.133 to 11.682, p<0.001) were independent good prognostic factors (table 2). With respect to OS, in univariate analysis, intrinsic subtype (HR=2.933, 95% CI 1.186 to 7.586, p=0.020), pathological response (HR=13.771, 95% CI 5.397 to 35.763, p<0.001), the rate of change in CD8^+^ TIL levels (HR=3.103, 95% CI 1.147 to 10.797, p=0.024), FOXP3^+^ TILs (HR=2.586, 95% CI 1.086 to 6.230, p=0.032) and the CFR (HR=8.279, 95% CI 2.800 to 35.365, p<0.001) were good prognostic factors. The rate of change in unstained TILs was not a significant prognostic factor (HR=1.316, 95% CI 0.568 to 3.191, p=0.524). Multivariate analysis revealed that TNBC subtype (HR=4.024, 95% CI 1.395 to 12.522, p=0.010), PR (HR=15.564, 95% CI 5.368 to 48.819, p<0.001) and a high rate of change in the CFR (HR=7.1877, 95% CI 1.921 to 34.687, p=0.003) had strong prognostic significance (table 3).

**Table 2:** Univariate and multivariate analyses with respect to recurrence-free survival in breast cancer subtypes

	Univariate analysis			Multivariate analysis		
	HR	95% CI	P values	HR	95% CI	P values
All breast cancer (n=129)						
Age (≤56 years)	1.406	0.729 to 2.755	0.309			
Tumour size (>2 cm)	1.098	0.434 to 3.693	0.859			
Lymph node (+)	2.004	0.895 to 5.342	0.095			
Ki-67 (>14)	1.012	0.523 to 1.974	0.971			
Subtype (TNBC)	1.494	0.553 to 4.706	0.441			
Pathological response (non-PR)	6.327	2.893 to 13.133	<0.001	5.260	2.373 to 11.145	<0.001
TIL (%) change (low)	1.276	0.656 to 2.536	0.473			
CD8 change (low)	3.114	1.430 to 7.773	0.003	2.304	1.052 to 5.776	0.036
FOXP3 change (high)	1.978	0.996 to 3.894	0.051			
CFR change (low)	5.612	2.581 to 14.001	<0.001	4.663	2.133 to 11.682	<0.001
TNBC (n=39)						
Age (≤56 years)	1.547	0.491 to 5.252	0.455			
Tumour size (>2 cm)	0.261	0.066 to 1.721	0.139			
Lymph node (+)	0.934	0.279 to 4.212	0.919			
Ki-67 (>14)	1.138	0.358 to 4.264	0.832			
Pathological response (non-PR)	25.642	6.873 to 123.724	<0.001	34.290	7.314 to 265.738	<0.001
TIL (%) change (low)	1.701	0.542 to 5.758	0.361			
CD8 change (low)	2.339	0.697 to 10.551	0.177			
FOXP3 change (high)	2.106	0.660 to 7.922	0.212			
CFR change (low)	11.420	2.215 to 208.742	0.002	13.021	2.241 to 258.136	0.002
HRBC (n=77)						
Age (≤56 years)	1.182	0.475 to 2.982	0.717			
Tumour size (>2 cm)	3.622	0.743 to 65.248	0.128			
Lymph node (+)	2.803	0.796 to 17.749	0.118			
Ki-67 (>14)	0.781	0.305 to 1.969	0.597			
Pathological response (non-PR)	2.132	0.488 to 6.625	0.277			
TIL (%) change (low)	1.069	0.404 to 2.885	0.892			
CD8 change (low)	3.167	1.134 to 11.196	0.027	2.746	0.976 to 9.741	0.056
FOXP3 change (high)	1.985	0.682 to 5.237	0.196			
CFR change (low)	4.740	1.779 to 14.833	0.002	4.377	1.641 to 13.712	0.003

Values in parentheses are 95% CIs.

CFR, CD8/FOXP3 ratio; FOXP3, forkhead box protein; HRBC, hormone receptor-positive breast cancer; PR, partial response; TIL, tumour-infiltrating lymphocyte; TNBC, triple-negative breast cancer.

**Table 3:** Univariate and multivariate analyses with respect to overall survival in breast cancer subtypes

	Univariate analysis			Multivariate analysis		
	HR	95% CI	P values	HR	95% CI	P values
All breast cancer (n=129)						
Age (≤56 years)	1.700	0.733 to 4.124	0.217			
Tumour size (>2 cm)	1.421	0.415 to 8.901	0.619			
Lymph node (+)	2.280	0.776 to 9.710	0.145			
Ki-67 (>14)	1.272	0.547 to 3.092	0.578			
Subtype (TNBC)	2.933	1.186 to 7.586	0.020	4.024	1.395 to 12.522	0.010
Pathological response (non-PR)	13.771	5.397 to 35.763	<0.001	15.564	5.368 to 48.819	<0.001
TIL (%) change (low)	1.316	0.568 to 3.191	0.524			
CD8 change (low)	3.103	1.147 to 10.797	0.024	2.271	0.769 to 8.352	0.143
FOXP3 change (high)	2.586	1.086 to 6.230	0.032	0.773	0.262 to 2.325	0.640
CFR change (low)	8.279	2.800 to 35.365	<0.001	7.177	1.921 to 34.687	0.003
TNBC (n=39)						
Age (≤56 years)	2.264	0.674 to 8.756	0.187			
Tumour size (>2 cm)	0.349	0.089 to 2.303	0.232			
Lymph node (+)	0.798	0.229 to 3.654	0.744			
Ki-67 (>14)	1.648	0.475 to 7.542	0.446			
Pathological response (non-PR)	11.812	0.043 to 23.141	<0.001	11.243	20.791 to 20.892	<0.001
TIL (%) change (low)	1.270	0.380 to 4.431	0.694			
CD8 change (low)	1.822	0.525 to 8.340	0.358			
FOXP3 change (high)	3.324	0.949 to 15.311	0.061			
CFR change (low)	9.847	1.883 to 180.764	0.004	8.346	1.538 to 155.128	0.010
HRBC (n=77)						
Age (≤56 years)	1.876	0.460 to 9.155	0.381			
Tumour size (>2 cm)	6.474	0.716 to 0.716	0.092			
Lymph node (+)	7.640	1.184 to 16.852	0.035	6.548	1.037 to 1.083	0.047
Ki-67 (>14)	0.955	0.225 to 4.054	0.948			
Pathological response (non-PR)	3.235	0.473 to 14.085	0.198			
TIL (%) change (low)	1.369	0.336 to 6.678	0.664			
CD8 change (low)	5.283	0.939 to 98.783	0.060			
FOXP3 change (high)	2.231	0.455 to 9.159	0.295			
CFR change (low)	11.081	1.969 to 207.323	0.004	10.333	1.832 to 193.336	0.006

Values in parentheses are 95% CIs.

CFR, CD8/FOXP3 ratio; FOXP3, forkhead box protein; HRBC, hormone receptor-positive breast cancer; PR, partial response; TIL, tumour-infiltrating lymphocyte; TNBC, triple-negative breast cancer.

### Prognostic value of changes in CD8^+^ TILs and FOXP3^+^ TILs before and after NAC in breast cancer subtypes

Additionally, we investigated the prognostic value of changes in the levels of CD8^+^ TILs and FOXP3^+^ TILs in each breast cancer subtype. Of the 39 patients with TNBC, pathological response (HR=25.642, 95% CI 6.873 to 123.724, p<0.001) and the rate of change in the CFR (HR=11.420, 95% CI 2.215 to 208.742 p=0.002) were significantly correlated with RFS in univariate analysis. Multivariate analysis showed that PR (HR=34.290, 95% CI 7.314 to 265.738, p<0.001) and a high rate of change in the CFR (HR=13.021, 95% CI 2.241 to 258.136, p=0.002) were independent prognostic factors for recurrence. Moreover, pathological response (HR=11.812, 95% CI 0.043 to 23.141, p<0.001) and the rate of change in the CFR (HR=9.847, 95% CI 1.883 to 180.764, p=0.004) were significantly correlated with OS in univariate analysis. Multivariate analysis showed that PR (HR=11.243, 95% CI 20.791 to 20.892, p<0.001) and a high rate of change in the CFR (HR=8.346, 95% CI 1.538 to 155.128, p=0.010) were independent prognostic factors for survival.

Of the 77 patients with hormone receptor-positive breast cancer (HRBC), the rate of change in CD8^+^ TIL levels (HR=3.167, 95% CI 1.134 to 11.196, p=0.027) and the CFR (HR=4.740, 95% CI 1.779 to 14.833, p=0.002) was significantly correlated with RFS in univariate analysis. Multivariate analysis revealed only a high rate of change in the CFR (HR=4.377, 95% CI 1.641 to 13.712, p=0.003) as an independent prognostic factor for recurrence. Additionally, lymph node status before NAC (HR=7.640, 95% CI 1.184 to 16.852, p=0.035) and the rate of change in the CFR (HR=11.081, 95% CI 1.969 to 207.323, p=0.004) were significantly correlated with OS in univariate analysis. Multivariate analysis showed that lymph node metastasis before NAC (HR=6.548, 95% CI 1.037 to 1.083, p=0.047) and a high rate of change in the CFR (HR=10.333, 95% CI 1.832 to 193.336, p=0.006) were independent prognostic factors for survival (tables 2 and 3). Since the number of patients with HER2BC was small (n=20), it could not be analysed.

## Discussion

In the present study, the proportion of unstained TILs, the number of CD8^+^ TILs and the CFR increased, and the number of FOXP3^+^ TILs decreased in breast tumours after NAC. Those results indicated that a regimen of FEC followed by paclitaxel±trastuzumab enhances antitumour immunity and reversal of immunoescape in cancer cells. This improvement in the immune microenvironment following NAC was significantly correlated with prognosis.

TILs are mononuclear immune cells in the tumour microenvironment. Infiltration of TILs before NAC is a useful biomarker to predict treatment response in patients with TNBC and HER2BC, two subtypes of highly malignant breast cancer.^11 30–33^ In these subtypes, high TILs group before NAC is significantly associated with higher pCR rate, good prognostic factor. In our study, investigating patients with non-pCR after NAC, the proportion of unstained TILs alone was not a useful predictor of outcome (online supplementary figure 3). Therefore, more detailed evaluation of TILs becomes necessary. In a previous study, Ladoire *et al* examined changes in the levels of CD8^+^ TILs and FOXP3^+^ TILs after NAC in 56 patients with breast cancer and reported that a high rate of change in the CFR was associated with pCR.^20^ Miyashita *et al* also analysed 78 patients with TNBC and reported that high rates of change in the level of CD8^+^ TILs and the CFR were significantly correlated with good RFS and OS.^15^ However, there have been few studies stratifying by intrinsic subtype of breast cancer. Our study is the first to indicate that a high rate of change in the CFR is an independent prognostic factor for good outcome in patients with TNBC and HRBC who do not achieve pCR after NAC.

10.1136/esmoopen-2017-000305.supp5 Supplementary file 5

TILs are mononuclear immune cells in the tumour microenvironment. Infiltration of TILs is a useful biomarker to predict treatment response in patients with TNBC and HER2BC, two subtypes of highly malignant breast cancer.^11 30–33^ These studies suggest that TNBC and HER2BC have high immunoactivity. However, based on the detailed subclassification of TILs as CD8^+^ TILs or FOXP3^+^ TILs, studies evaluating the prognostic significance of CD8^+^ TILs or FOXP3^+^ TILs or the CFR in the intrinsic molecular subtypes of breast cancer have shown conflicting results.^15 17 34–40^ One possible explanation consistent with these discrepant findings is that HRBC is also considered to be associated with some kind of immunity.

The CFR reflects the interplay between CD8^+^ TILs and Treg cells in a tumour and indicates the activity of the immune microenvironment. A higher CFR has been shown to be significantly associated with better survival in hormone receptor-negative tumours.^15–17^ In the present study, we focused on the rate of changes in the CFR induced by NAC and demonstrated that an increase in the CFR was significantly associated with improved clinical outcomes in not only TNBC but also HRBC. This result suggests that the change in the CFR after NAC may be a more accurate indicator of immune activity induced by chemotherapy.

The recently identified immune checkpoint markers, programmed death-1 (PD-1) and programmed death-ligand 1 (PD-L1), are present in some breast cancers.^41^ The PD-1/PD-L1 axis, a major immune checkpoint pathway, leads to a reduction in the immune response by inducing T cell tolerance.^42^ In previous studies, Muenst *et al* reported that PD-1^+^ TILs are significantly associated with worse OS in the luminal B and basal-like subtypes.^43^ Ali *et al* also reported that PD-L1 expression is significantly enriched in the basal-like subtype and is correlated to the presence of TILs.^44^ In addition, one recent study indicated that PD-L1 expression in residual tumours is significantly associated with the levels of CD8^+^ TILs and FOXP3^+^ TILs and may be a useful prognostic marker in patients with breast cancer following NAC.^45^ These results suggest that by further evaluating the changes in other TILs, such as PD-1^+^ TILs, along with those in CD8^+^ TILs and FOXP3^+^ TILs in patients treated with NAC, more accurate identification of patient-specific immune mechanisms and prediction of prognosis may be possible.

As a potential limitation, since the sample size of our study was small, we did not evaluate the relationship between HER2BC and the rate of change in the levels of CD8^+^ TILs and FOXP3^+^ TILs or the CFR. In addition, we analysed without dividing patients with HRBC into a HER2-positive and HER2-negative group.

This is the first study to indicate that improvement in the immune microenvironment following NAC has a relationship with good outcome, and that a high rate of change in the CFR could be a potential prognostic marker in patients with TNBC and HRBC who do not achieve pCR after NAC.
