# Supplementary material for: Predictive value of improvement in the immune tumour microenvironment in patients with breast cancer treated with neoadjuvant chemotherapy
Source: ESMO Open. 2018 Aug 30;3(6):e000305. doi: 10.1136/esmoopen-2017-000305 (PMC6135412; doi:10.1136/esmoopen-2017-000305)
Supplement: Supplementary file 1 [file esmoopen-2017-000305supp001.pdf]

Supplementary Table 1. Correlation between clinicopathological features and CD8^{+} TILs, FOXP3^{+} TILs, and the CD8/FOXP3 ratio before neoadjuvant chemotherapy.

Parameters	CD8^{+} TILs			FOXP3^{+} TILs			CD8/FOXP3		
	Low (n = 71)	High (n = 64)	p value	Low (n = 63)	High (n = 71)	p value	Low (n = 72)	High (n = 62)	p value
Age at operation									
≤56	31 (43.6 %)	35 (54.7 %)		27 (42.9 %)	38 (53.5 %)		33 (45.8 %)	32 (51.6 %)	
>56	40 (56.4 %)	29 (45.3 %)	0.230	36 (57.1 %)	33 (46.5 %)	0.231	39 (54.2 %)	30 (48.4 %)	0.603
Tumor size									
≤2 cm	10 (14.1 %)	7 (10.9 %)		9 (14.3 %)	8 (11.3 %)		8 (11.1 %)	9 (14.5 %)	
>2 cm	61 (85.9 %)	57 (89.1 %)	0.614	54 (85.7 %)	63 (88.7 %)	0.615	64 (88.9 %)	53 (85.5 %)	0.609
Lymph node status									
Negative	16 (22.5 %)	23 (35.9 %)		15 (23.8 %)	23 (32.4 %)		20 (27.8 %)	18 (32.4 %)	
Positive	55 (77.5 %)	41 (64.1 %)	0.092	48 (76.2 %)	48 (67.6 %)	0.338	52 (72.2 %)	44 (67.6 %)	0.872
Ki67									
≤14 %	35 (49.3 %)	30 (46.9 %)		23 (36.5 %)	41 (57.7 %)		39 (54.2 %)	25 (40.3 %)	
>14 %	36 (50.7 %)	34 (53.1 %)	0.863	40 (63.5 %)	30 (42.3 %)	0.016	33 (45.8 %)	37 (59.7 %)	0.122
Intrinsic subtype									
TNBC	20 (28.2 %)	19 (29.7 %)		21 (33.3 %)	18 (25.3 %)		19 (26.4 %)	20 (32.3 %)	
HER2BC	8 (11.3 %)	10 (15.6 %)		9 (14.3 %)	9 (12.7 %)		9 (12.5 %)	9 (14.5 %)	
HRBC	43 (60.5 %)	35 (54.7 %)	0.702	33 (52.4 %)	44 (62.0 %)	0.514	44 (61.1 %)	33 (53.2 %)	0.652
Pathological response									
non-PR	8 (11.3 %)	10 (15.6 %)		6 (9.5 %)	12 (16.9 %)		13 (18.1 %)	5 (8.1 %)	
PR	63 (88.7 %)	54 (84.4 %)	0.613	57 (90.5 %)	59 (83.1 %)	0.310	59 (81.9 %)	57 (91.9 %)	0.127
TILs (%)									
≤10 %	48 (67.6 %)	29 (45.3 %)		27 (42.9 %)	49 (69.0 %)		53 (73.6 %)	23 (37.1 %)	
>10 %	23 (32.4 %)	35 (54.7 %)	0.015	36 (57.1 %)	22 (31.0 %)	0.003	19 (26.4 %)	39 (62.9 %)	<0.001
CD8^{+}TILs									
Low	Not determined	Not determined		45 (71.4 %)	25 (35.2 %)		47 (65.3 %)	23 (37.1 %)	
High				18 (28.6 %)	46 (64.8 %)	<0.001	25 (34.7 %)	39 (62.9 %)	0.002
FOXP3^{+}TILs									
Low	46 (64.8 %)	18 (28.1 %)		Not determined	Not determined		22 (30.6 %)	41 (66.1 %)	
High	25 (35.2 %)	46 (71.9 %)	<0.001				50 (69.4 %)	21 (33.9 %)	<0.001
CD8^{+}/FOXP3^{+}									
Low	48 (67.6 %)	25 (39.1 %)		22 (34.9 %)	50 (70.4 %)		Not determined	Not determined	
High	23 (32.4 %)	39 (60.9 %)	<0.001	41 (65.1 %)	21 (29.6 %)	<0.001			

TILs, tumor-infiltrating lymphocytes. FOXP3, forkhead box protein. TNBC, triple-negative breast cancer. HER2BC, human epidermal growth factor receptor 2-enriched breast cancer. HRBC, hormone receptor-positive breast cancer. PR, partial response.
