# Supplementary figures and images for: Predictive value of improvement in the immune tumour microenvironment in patients with breast cancer treated with neoadjuvant chemotherapy
Source: ESMO Open. 2018 Aug 30;3(6):e000305. doi: 10.1136/esmoopen-2017-000305 (PMC6135412; doi:10.1136/esmoopen-2017-000305)

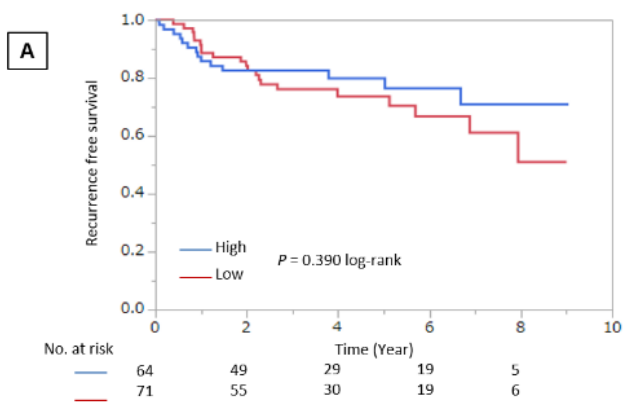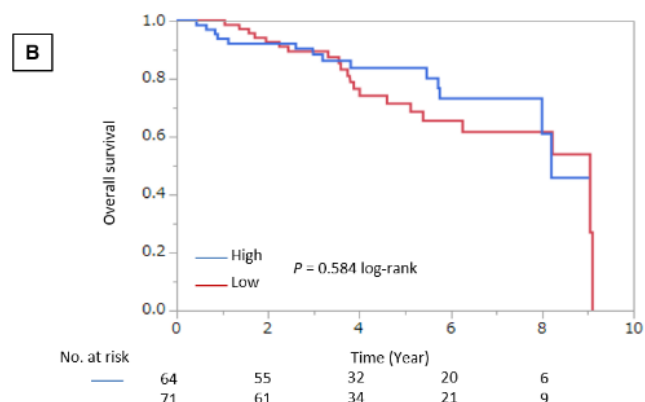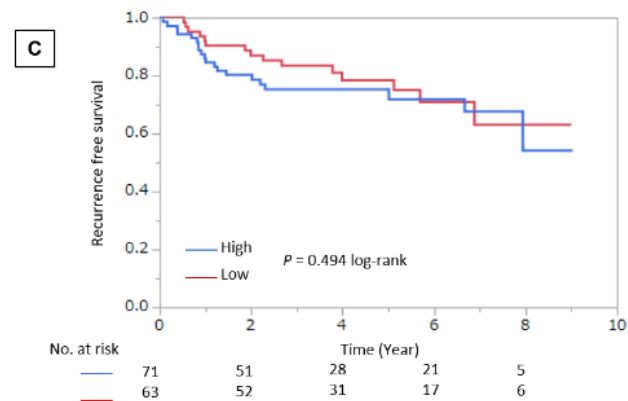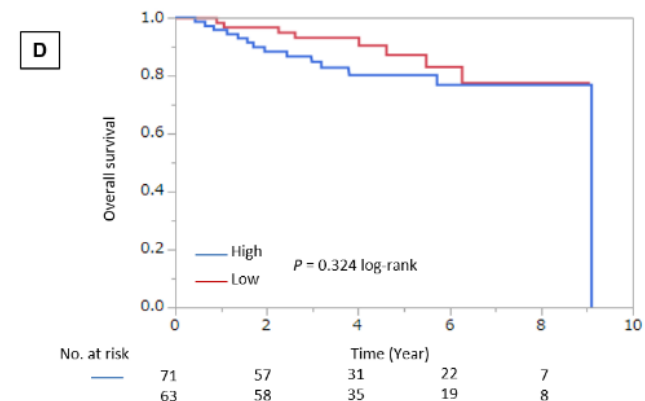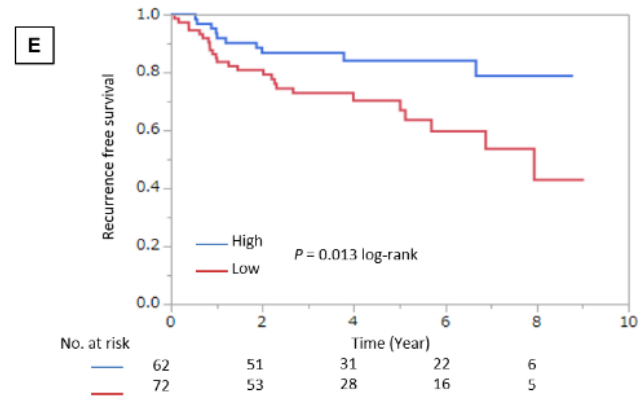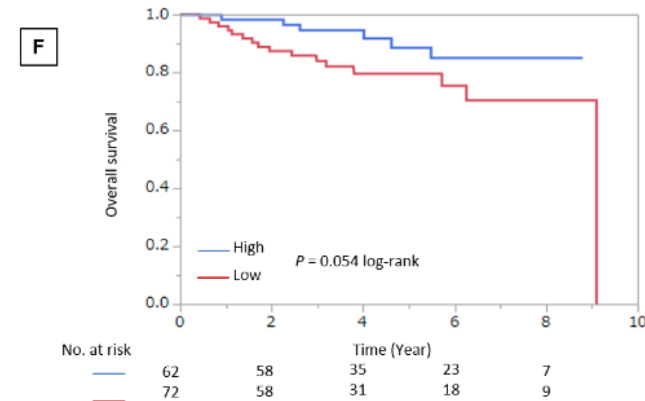

Supplement: Supplementary file 2 [file esmoopen-2017-000305supp002.pdf]

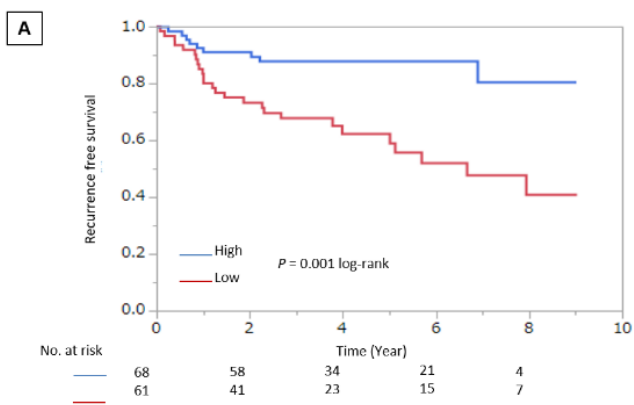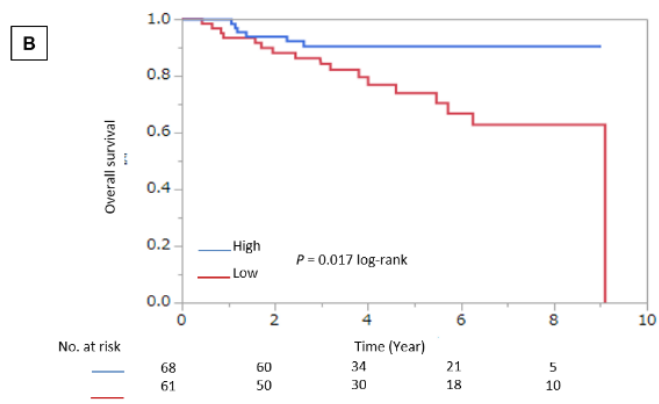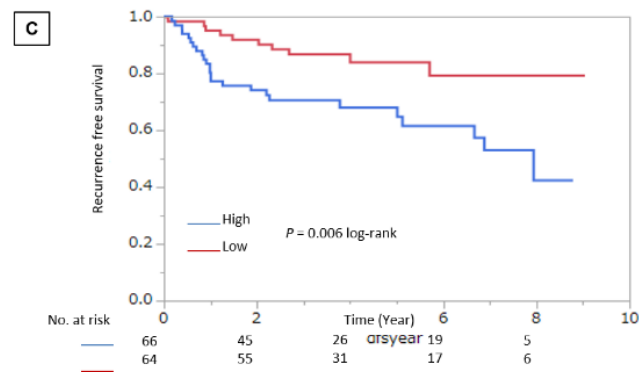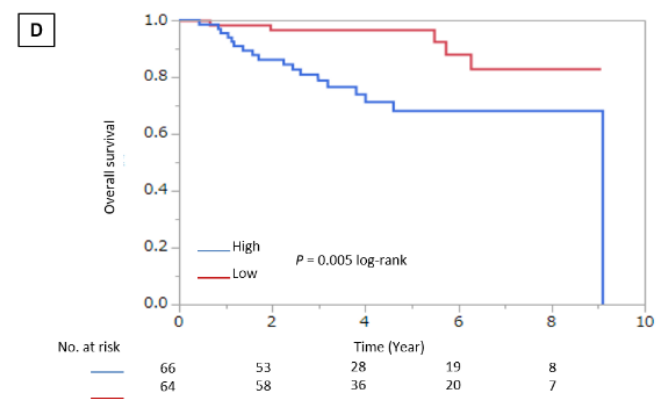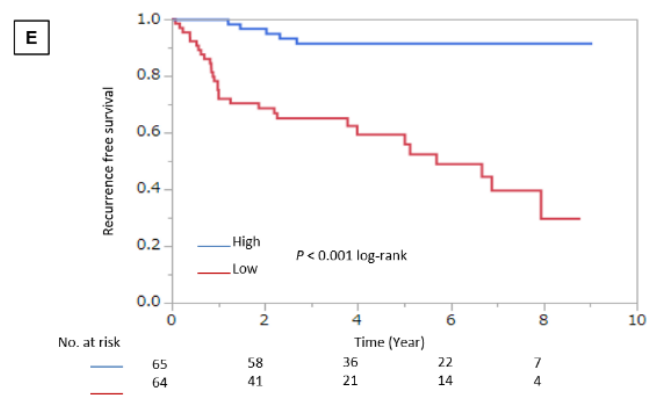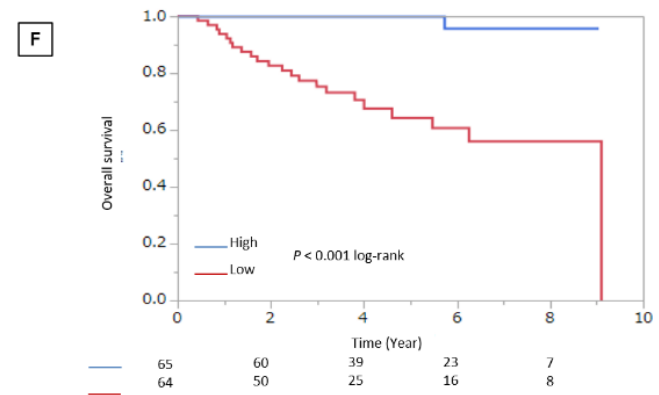

Supplement: Supplementary file 4 [file esmoopen-2017-000305supp004.pdf]

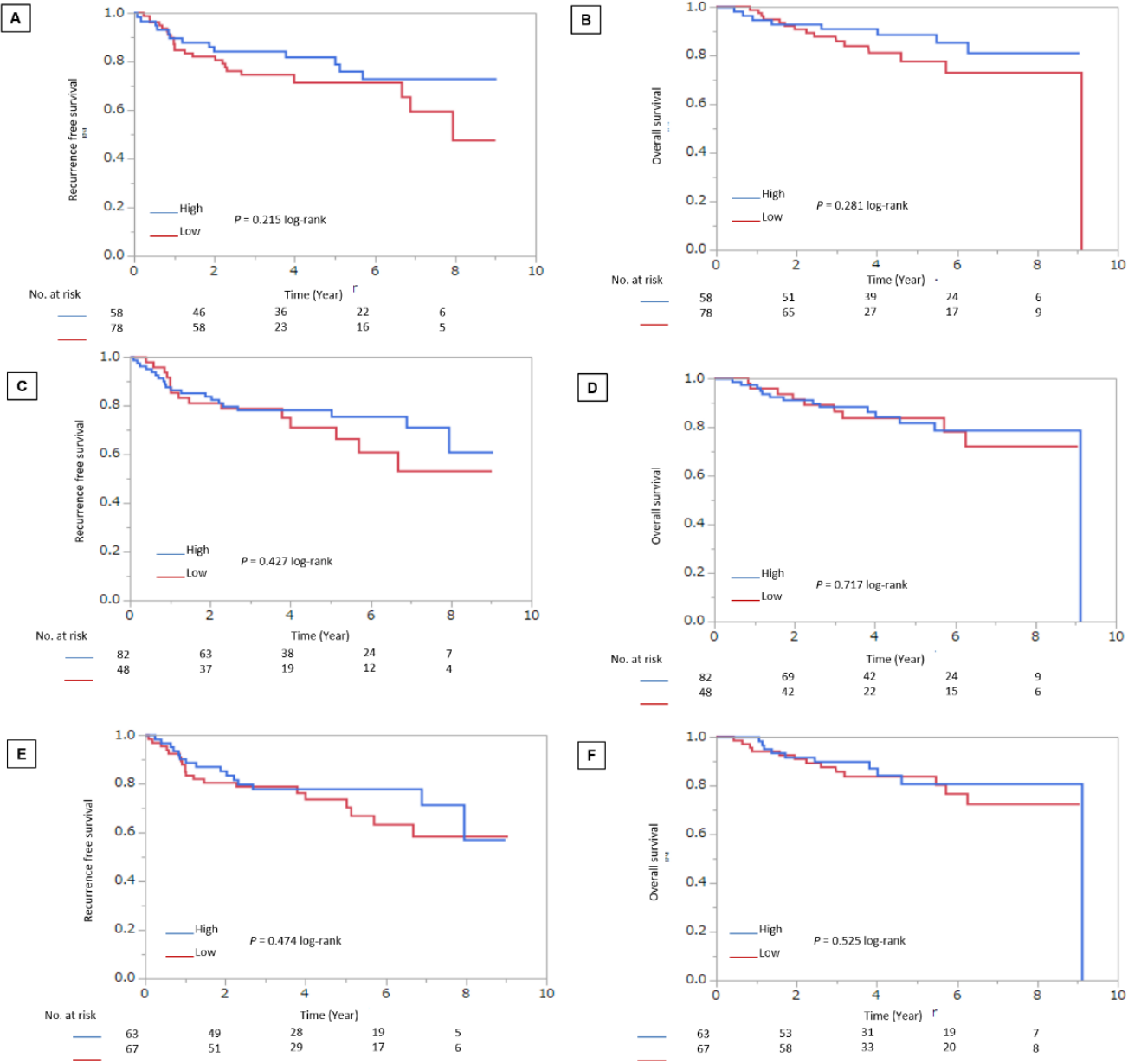

Supplement: Supplementary file 5 [file esmoopen-2017-000305supp005.pdf]
