# Supplementary material for: Predictive value of improvement in the immune tumour microenvironment in patients with breast cancer treated with neoadjuvant chemotherapy
Source: ESMO Open. 2018 Aug 30;3(6):e000305. doi: 10.1136/esmoopen-2017-000305 (PMC6135412; doi:10.1136/esmoopen-2017-000305)
Supplement: Supplementary file 3 [file esmoopen-2017-000305supp003.pdf]

Supplementary Table 2. Correlation between clinicopathological features and CD8$^+$ TILs, FOXP3$^+$ TILs, and the CD8/FOXP3 ratio after neoadjuvant chemotherapy.

Parameters	CD8$^+$ TILs			FOXP3$^+$ TILs			CD8/FOXP3		
	Low (n = 61)	High (n = 68)	p value	Low (n = 64)	High (n = 66)	p value	Low (n = 64)	High (n = 65)	p value
Age at operation									
≤56	24 (39.3 %)	40 (58.8 %)		25 (39.1 %)	39 (59.1 %)		34 (53.1 %)	30 (46.2 %)	
>56	37 (60.7 %)	28 (41.2 %)	0.035	39 (60.9 %)	27 (40.9 %)	0.024	30 (46.9 %)	35 (53.8 %)	0.483
Tumor size									
≤2 cm	124 (39.3 %)	4 (5.9 %)		11 (17.2 %)	5 (7.9 %)		8 (12.5 %)	8 (12.3 %)	
>2 cm	37 (60.7 %)	64 (94.1 %)	0.030	53 (82.8 %)	61 (92.1 %)	0.114	56 (87.5 %)	57 (87.7 %)	0.974
Lymph node status									
Negative	13 (21.3 %)	25 (36.8 %)		22 (34.4 %)	16 (24.2 %)		16 (25.0 %)	22 (33.8 %)	
Positive	48 (78.7 %)	43 (63.2 %)	0.081	42 (65.6 %)	50 (75.8 %)	0.249	48 (75.0 %)	43 (66.2 %)	0.335
Ki67									
≤14 %	31 (50.8 %)	30 (44.1 %)		32 (50.0 %)	30 (45.5 %)		31 (48.4 %)	30 (46.2 %)	
>14 %	30 (49.2 %)	38 (55.9 %)	0.483	32 (50.0 %)	36 (54.5 %)	0.726	33 (51.6 %)	35 (53.8 %)	0.861
Intrinsic subtype									
TNBC	17 (27.9 %)	21 (30.9 %)		16 (25.0 %)	22 (33.3 %)		23 (35.9 %)	15 (23.1 %)	
HER2BC	7 (11.5 %)	11 (16.2 %)		9 (14.1 %)	9 (13.7 %)		9 (14.1 %)	9 (13.8 %)	
HRBC	37 (60.6 %)	36 (52.9 %)	0.623	39 (60.9 %)	35 (53.0 %)	0.566	32 (50.0 %)	41 (63.1 %)	0.246
Pathological response									
non-PR	11 (18.0 %)	7 (10.3 %)		6 (9.4 %)	12 (18.2 %)		14 (21.9 %)	4 (6.1 %)	
PR	50 (82.0 %)	61 (89.7 %)	0.309	58 (90.6 %)	54 (81.8 %)	0.205	50 (78.1 %)	61 (93.9 %)	0.012
TILs (%)									
≤10 %	36 (59.0 %)	12 (17.7 %)		35 (54.7 %)	13 (19.7 %)		21 (32.8 %)	27 (41.5 %)	
>10 %	25 (41.0 %)	56 (82.3 %)	<0.001	29 (45.3 %)	53 (80.3 %)	<0.001	43 (67.2 %)	38 (58.5 %)	0.364
CD8$^+$TILs									
Low	Not determined	Not determined		38 (59.4 %)	24 (36.4 %)		34 (53.1 %)	27 (41.5 %)	
High				26 (40.6 %)	42 (63.6 %)	0.014	30 (46.9 %)	38 (58.5 %)	0.219
FOXP3$^+$TILs									
Low	37 (60.7 %)	26 (38.2 %)		Not determined	Not determined		10 (15.6 %)	53 (81.5 %)	
High	24 (39.3 %)	42 (61.8 %)	0.014				54 (84.4 %)	12 (18.5 %)	<0.001
CD8$^+$/FOXP3$^+$									
Low	34 (55.7 %)	30 (44.1 %)		10 (15.6 %)	54 (81.8 %)		Not determined	Not determined	
High	27 (44.3 %)	38 (55.9 %)	0.219	54 (84.4 %)	12 (18.2 %)	<0.001			

TILs, tumor-infiltrating lymphocytes. FOXP3, forkhead box protein. TNBC, triple-negative breast cancer. HER2BC, human epidermal growth factor receptor 2-enriched breast cancer. HRBC, hormone receptor-positive breast cancer. PR, partial response.
